# The impact of digital economy on environmental pollution: Evidence from 267 cities in China

**DOI:** 10.1371/journal.pone.0297009

**Published:** 2024-01-26

**Authors:** Honglin Yuan, Jia Liu, Xiaona Li, Shen Zhong

**Affiliations:** 1 Jiangxi University of Finance and Economics, Nanchang, China; 2 Harbin University of Commerce, Harbin, China; East China Normal University, CHINA

## Abstract

Environmental pollution has become a pressing global issue, severely threatening human health and ecosystems. As an emerging driver of economic development in countries worldwide, the digital economy (DE) has the potential to enhance resource utilization efficiency and promote the development of clean technologies, thereby reducing environmental pollution. Based on the panel data of 267 cities in China from 2012 to 2021, the spatial econometric model is used to test the impact of DE on environmental pollution. The mediating effect model is used to explore the transmission mechanism of DE affecting environmental pollution. The panel threshold model is used to examine the threshold effect of marketization. The results are as follows: (1) DE can significantly reduce environmental pollution. The conclusion is still valid after conducting robustness tests such as selecting historical data as instrumental variables and the “Broadband China” pilot as a quasi-natural experiment. (2) From the perspective of transmission mechanism, DE can reduce environmental pollution through green technology innovation and industrial structure upgrading. (3) From the perspective of spatial spillover effect, DE can reduce the environmental pollution level of surrounding cities. (4) From the perspective of threshold effect, DE has obvious market-oriented single threshold effect on environmental pollution. When the marketization level crosses the threshold of 11.6611, the emission reduction effect of DE is significant. (5) From the perspective of heterogeneity, DE has a heterogeneous impact on environmental pollution in cities with different geographic locations, resource endowments and administrative level. Regarding geographical heterogeneity, DE can effectively reduce environmental pollution in eastern and central regions, but has no significant impact on environmental pollution in western regions. Regarding the heterogeneity of resource endowment, compared to non-resource-based cities, resource-based cities suffer more from the negative effects of DE on their environment. Regarding the heterogeneity of administrative levels, compared with non-central cities, the DE of central cities has a greater emission reduction effect. Based on empirical results, this paper proposes strategic recommendations in areas such as enhancing the application of DE in emission reduction, upgrading industrial structures, promoting green technology innovation, and improving the level of marketization. This study not only enriches the research of DE and environmental pollution, but also provides a reference for the formulation of environmental pollution control policies.

## 1 Introduction

There has been rapid growth in China’s economy in recent years [1, 2]. Statistics show that China’s per capita GDP in 2022 is 12,251 US dollars, surpassing the world’s per capita GDP level. However, the extensive economic development mode which excessively relies on the environment has brought about serious environmental pollution problems [3]. In order to address the increasingly serious environmental problems, the Chinese government has issued a series of environmental protection policies, which have achieved remarkable results. But there is still a significant gap with the expected level. According to the Global Environmental Performance Index Report released by Yale University in 2022, the score of China’s environmental performance Index is 28.4, ranks 160th out of 180 participating countries, which indicates that China’s environmental problems are still very serious [4]. In this context, how to reduce environmental pollution and achieve sustainable economic development will become the focus of China in the future.

For the purpose of reducing environmental pollution, scholars have discussed the impact of economic development [5], technological progress [6], urbanization [7], population [8], transportation [9], energy consumption [10] and other economic and social factors on environmental pollution. However, with the steady development of economy, these factors can no longer be the main means to solve the problem of environmental pollution. As an important focus for China to replace old growth drivers with new ones, DE has been deeply integrated into all sectors of the economy and society. It contributes to the resource allocation and innovation efficiency [11]. Therefore, the effect of DE on environmental pollution is worthy of attention. For one thing, DE can promote the transfer of labor-intensive industrial structure to technology-intensive and environment-friendly industrial structure [12]. For another, DE can reduce transaction costs by reducing information asymmetry [13] and provide financial assistance to enterprises to develop green technologies. In addition, marketization can promote the multi-directional flow of factors (such as information and data) related to DE, thus affecting the role of DE in the environment. Therefore, exploring the transmission mechanism of DE affecting environmental pollution and the role of marketization in the relationship between the two is of great significance for realizing emission reduction driven by DE.

Firstly, based on the panel data of 267 cities from 2012 to 2021, this paper uses the spatial Durbin model (SDM) and mediating effect model to empirically test the impact of DE on environmental pollution and its transmission mechanism. Secondly, taking the degree of marketization as threshold variable, this paper uses the panel threshold model to deeply explore the role of marketization in the relationship between DE and environmental pollution. Finally, considering the differences in geographical location, resource endowment and administrative level, this paper deeply examines the heterogeneous impact of DE on environmental pollution in cities with different geographical location, resource endowment and administrative level.

Existing research focuses on the surface relationship between the DE and environmental pollution, lacking depth into its mechanisms and threshold effects, and overlooking regional differences in sample selection. This limits a comprehensive understanding of their complex relationship. This paper aims to explore the impact and mechanisms of the DE on environmental pollution. It also seeks to identify the variations of this impact among different cities, filling the gaps in existing research. Specifically, the contributions of this paper are reflected in the following aspects: Theoretically, explaining the mechanisms, threshold effects, and spatial spillover effects of how the DE impacts environmental pollution. Compared to existing researches, it offers a more comprehensive perspective on the theoretical dimension. Based on this, this paper uses the SDM to empirically test the impact of the DE on environmental pollution, yielding more accurate estimates compared to existing research. Additionally, this paper thoroughly examines the heterogeneous effects of DE on environmental pollution across cities with different geographical locations, resource endowments, and administrative levels. This insight is crucial for policymakers to design policies that are well-suited to individual regional contexts.

**Fig 1:**
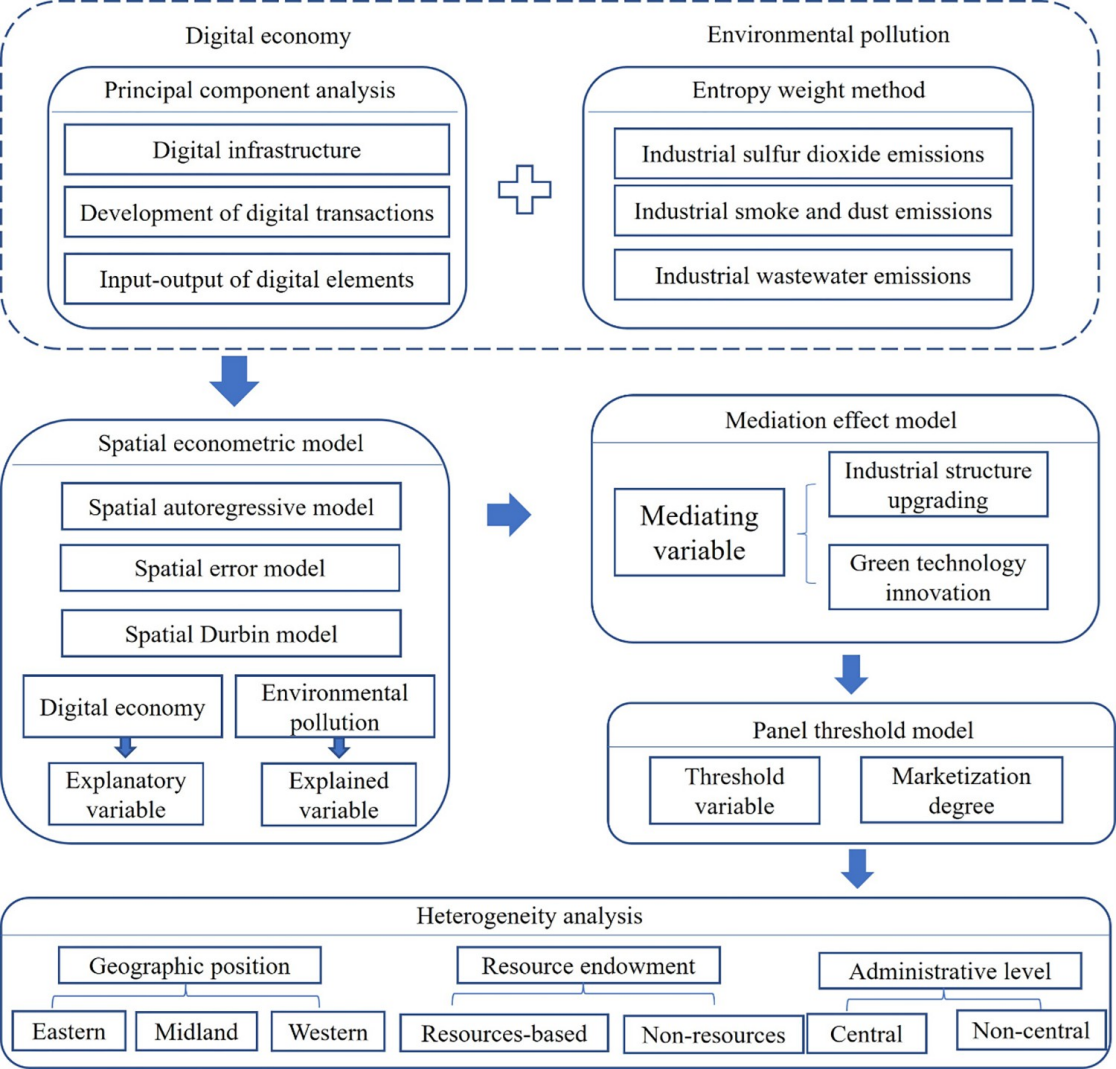
Research framework.

The remainder of this paper is organized as follows: In the second part, a literature review is presented. In the third part, the research hypothesis is presented. In the fourth part, data and methods are discussed. In the fifth part, empirical findings are presented. The sixth part is the discussion of results. The seventh part is the results and policy recommendations. The research framework is shown in Fig 1.

## 2 Literature review

### 2.1 Environmental pollution

#### 2.1.1 Measurement of environmental pollution

Regarding the measurement of intensity of environmental pollution, most scholars currently utilize a single indicator to assess the degree of environmental pollution. Taking sulfur dioxide as an evaluation index of environmental pollution, Zhang et al. [14] studied the dynamic effect of industrial co-agglomeration on environmental pollution. Zhu et al. [15], Wang et al. [16], Xu et al. [17] studied the relationship between FDI and sulfur dioxide emissions. Some scholars took industrial soot emissions as an evaluation index of environmental pollution [18, 19]. In addition, PM2.5 concentration can also be used to evaluate environmental pollution [20]. However, a single index cannot fully and scientifically reflect environmental pollution [21].

#### 2.1.2 Influencing factors of environmental pollution

There are many researches on the factors that influence environmental pollution. Some scholars discussed the factors that influence environmental pollution from the perspective of technological progress. For example, Ahma et al. [22] believed that technological innovation could promote economic development and relax environmental pollution at the same time, thus achieving sustainable economic growth. Wang et al. [23] found that green total factor productivity was significantly promoted by technological innovation of renewable energy. Liang et al. [24] found that logistics technology innovation had a rebound effect on carbon emissions, which can increase carbon dioxide emissions. Some scholars have discussed the influencing factors of environmental pollution from the standpoint of international trade. For example, Adeel-Farooq et al. [25] found that the influence of FDI on the environmental performance of the host country mainly depended on the policy environment of the source country. Sapkota and Bastola [26] found that FDI could reduce environmental pollution, supporting the “pollution haven hypothesis”. Some scholars have discussed the factors affecting environmental pollution from a policy perspective. For instance, Yan et al. [27] used DID to empirically test the emission reduction effect of China’s emission trading pilot policy. They found that the pilot emissions trading policy reduced haze pollution. Some scholars have found that energy consumption can also impact the environment. For instance, Wang [28] empirically examined the impact of biomass energy consumption on the environment using panel data of BRICS countries from 1992 to 2013. The study found that biomass energy consumption can alleviate environmental pressure. In addition, environmental regulation [29] and political promotion championships [30] have an impact on environmental pollution.

### 2.2 DE and environmental pollution

Scholars have done lots of researches on the economic and social impact of DE. From a micro perspective, relying on advanced digital technology, both digital finance and DE can reduce the degree of information asymmetry and ease the financing constraints of enterprises [31]. From a macro perspective, DE can promote economic development [32]. Besides, DE also has a significant role in playing in international trade [33].

With the rise of DE, there has been considerable interest of many scholars in DE’s potential role in environmental protection. At present, there is still some controversy about the influence of DE on environmental pollution. Using the fixed effect model, Dong et al. [34] found that the DE significantly reduced carbon emissions. Compared with PM2.5 and NO_2_ concentration, the DE had the strongest inhibition effect on the concentration of SO_2_. Using a dynamic panel model, Wu et al. [35] also found that DE had a negative impact on air pollution. Taking 30 provinces in China as a research sample, Ma et al. [36] used panel data model to analyze the impact of digitalization on carbon emissions and found that digitalization can suppress CO_2_ emissions. Similarly, using the panel data model, Deng et al. [37] explored the impact of DE on the green total factor productivity of manufacturing enterprises. They found that DE positively impacted green total factor productivity in manufacturing. However, the negative effect of DE on quality of environment cannot be ignored. Using data from 73 countries and the modified ordinary least squares method, Danish et al. [38] found that DE increased carbon emissions in low-income countries. Although the above studies have successfully estimated the overall impact of DE on environmental pollution, they have not revealed the mechanism behind it. In addition, these studies do not consider the spatial spillover effect of DE on environmental pollution, which may be the reason for the uncertainty of results.

### 2.3 Spatial econometric model

At present, the spatial econometric model is widely used to investigate the spatial correlation between variables. In terms of spatial correlation of environmental pollution, Du et al. [39] found that PM2.5 concentration had a significant spatial dependence within an area of 200km^2^. Jiang et al. [40] utilized the spatial econometric model to investigate the factors that influenced SO_2_ emission. He found that SO_2_ emission had a positive spatial correlation. Cheng [41], Que et al. [42] and Zhao et al. [43] all found that environmental pollution had a significant spatial correlation, which indicates that environmental governance needs joint efforts among regions. Meanwhile, some researchers have studied the spatial spillover effect of DE [44].

The above literature offers a foundation for the research in this paper, there are still some limitations. Firstly, in theory, most literature analyzed the overall impact of DE on environmental pollution, but did not deeply analyze the mechanism of DE’s impact on environmental pollution. Secondly, in research methods, little literature includes DE into spatial econometric models to investigate the spatial spillover effects of DE on environmental pollution. Thirdly, in terms of sample selection, most existing literature selects panel data of provincial or several cities as research samples, which ignores the differences between regions.

For complementing existing literature, this paper makes the following contributions: Firstly, theoretically, this paper analyzes the effect mechanism of DE on environmental pollution from the two perspectives of industrial structure upgrading and green technology innovation. Simultaneously, incorporating the degree of marketization into analysis framework, this paper analyzes the role of marketization in the process of DE affecting environmental pollution. Secondly, in terms of research methods, this study empirically analyzes the impact of DE on environmental pollution by using SDM. Compared with other models, SDM has the following advantages: Firstly, the SDM considers the spatial spillover effect. Using this model, we can more accurately assess the effectiveness of DE in reducing environmental pollution in local and surrounding areas. Secondly, SDM can provide robust estimation results and avoid the bias caused by spatial autocorrelation, so as to ensure the reliability and accuracy of research findings. On this basis, this paper conducts an exogenous shock test with the pilot policy of “Broadband China”. It can solve the potential endogenous problems, so as to better prove the robustness of results. Thirdly, in terms of sample selection, panel data on 267 cities from 2012 to 2021 is used for this study, which is conducive to decision-makers to formulate relevant policies according to the actual situation.

## 3 Research hypothesis

With the natural advantages of cross-temporal information dissemination, data sharing and transaction cost reduction, DE can not only directly affect environmental pollution, but also indirectly affect environmental pollution by industrial structure upgrading and green technology innovation. In addition, the acceleration of marketization process has a certain impact on the emission reduction effect of DE. This paper primarily examines the direct impact of DE on environmental pollution, exploring its transmission mechanism, threshold effect, and spatial spillover effect.

### 3.1 The direct effect of DE on environmental pollution

DE is a new economic model centered around data as the core production element, information and communication technology as the core driving force, and modern information networks as important carriers [45]. With the rapid development of digital technologies, the network effects brought by DE have profoundly changed economic and social development. The theory of network effects refers to the increase in value of a product or service as the number of users using that product or service increases [46]. This paper extends the application field of this theory from the socio-economic domain to the environmental domain, analyzing the direct impact of DE on environmental pollution from the following three aspects:

Firstly, the DE, through the Internet, social media, and other online platforms, connects a vast number of users. This extensive connectivity generates a strong network effect, allowing environmental information to spread quickly. It enhances people’s environmental awareness, promoting more eco-friendly consumption and lifestyles [47]. Secondly, in the context of DE, consumers express their demand for eco-friendly products and services through various online channels. Companies can swiftly modify their strategies to create products that meet market and environmental requirements upon receiving this feedback. This real-time feedback mechanism provides support for the achievement of environmental goals. Thirdly, the network effect of DE also helps governments enforce environmental policies more effectively. Public engagement in real-time policy updates fosters an open policy-making environment and enhances the efficiency of implementing environmental policies.

**Hypothesis 1:** DE can reduce environmental pollution.

### 3.2 The transmission mechanism of DE affecting environmental pollution

In this section, we explore in detail how the DE reduces environmental pollution by upgrading industrial structures and innovating green technologies, aiding in enhancing our understanding of the logical relationship between DE and environmental pollution.

DE facilitates the reallocation of production factors towards capital and technology-intensive industries [48], promoting industrial structure upgrading. In more advanced economic structures, high-tech industries often take a dominant position, such as information technology, biotechnology, etc. Compared to less developed economic structures, these industries tend to have higher resource use efficiency and lower pollution emissions [49].

DE can promote technological innovation in several ways: Firstly, as an important application of DE, digital finance can boost enterprises’ green tech innovation by funding their R&D activities [50]. Secondly, integrating digital technologies with physical enterprises, DE can drive new green innovations using AI analysis systems and advanced sensors. Thirdly, DE can provide a communication platform for innovative entities through big data and the Internet of things, so as to realize resource sharing and complementary advantages [51]. The degree of green technology innovation increases. Green technology enhances energy efficiency and pollution control, reducing environmental pollution.

**Hypothesis 2:** DE can reduce pollution through industrial structure upgrading and green technology innovation.

### 3.3 The threshold effect of DE on environmental pollution

In this section, we delve deeply into how the level of marketization impacts the environmental effects of DE. We analyze the roles of market openness, competitive incentives, and information dissemination in mitigating environmental pollution at different levels of marketization. This exploration uncovers the correlation between the degree of marketization and DE in promoting environmental protection. Such insights hold significant value for understanding the influence of DE on environmental pollution at various stages of marketization.

The degree of marketization mainly affects the effect of DE on environmental pollution from the following three aspects: Firstly, the improvement of marketization can break the barriers to entry and exit of the digital industry. Production factors can flow freely, which produces economies of scale and improve production efficiency. The elimination of backward production capacity is accelerated, resulting in lots of low pollution and low emission enterprises. Secondly, while promoting a more unified and standardized market order, the improvement of marketization intensifies market competition. In the fierce market competition, enterprises invest more in green technology innovation, adopting clean, efficient production technologies to reduce emissions. Thirdly, in the DE, the transmission and exchange of information is convenient, but the field of transmission is relatively single. Improving marketization can promote a multi-directional flow of information and enhance the demonstrative effect of pollution control.

**Hypothesis 3:** Giving full play to the role of market is conducive to enhancing the effect of DE on reducing environmental pollution.

### 3.4 The spatial spillover effect of DE on environmental pollution

The spatial spillover effect of DE on environmental pollution cannot be neglected. In this section, we deeply study the interaction between regional economy and technology, and explore how DE achieves its cross-regional impact on environmental pollution by promoting the cross-regional flow of technology and information.

Firstly, based on the theory of technological spillover [52], DE can reduce spatial boundaries and promote the spillover of knowledge and technology, thus improving the green technology innovation capacity of the surrounding areas. Secondly, DE can promote the free flow of production factors like capital and labor, so as to provide a material guarantee for green innovation activities in surrounding areas. Environmental pollution levels in surrounding areas are reduced [53]. Thirdly, DE enhances collaboration and competition among neighboring regional governments, aiding in the reduction of environmental pollution.

**Hypothesis 4:** The DE in a region can reduce environmental pollution in the surrounding regions.

## 4 Research methods and data

### 4.1 Baseline regression model

This paper constructs a baseline regression model to analyze the impact of DE on environmental pollution. The specific formula is as follows:

$$\ln EP_{\mathrm{it}}=\eta_{0}+\eta_{1}DE_{it}+\eta_{2}\sum X_{it}^{'}+\mu_{i}+\gamma_{t}+\varepsilon_{it}$$ (1)

Where, *i* represents the city, *t* represents time. *lnEP* represents the logarithm of environmental pollution. *DE* is the core explanatory variable digital economy. *μ* is the individual fixed effect. *γ* is the time fixed effect. *ε* is the random error term.

The assumptions of this model mainly include: Firstly, each individual and time point has its own unique characteristics, which affects the explained variables. Secondly, there is no multicollinearity problem. Thirdly, the error term should be independent and normal distribution, and has a constant variance, no autocorrelation and heteroscedasticity.

The model is suitable for panel data. When there is individual and time heterogeneity in the data, this model can control the unobservable individual and time effects.

### 4.2 Spatial econometric model

Neighboring regions in China have similar economic structures and industrial models, leading to a strong correlation in their pollution emissions. Meanwhile, DE often has an impact on neighboring regions. Therefore, when studying the effect of DE on environmental pollution, ignoring the spatial effect may lead to unrobust results. In this paper, SDM is used for empirical analysis. Its model can be set to:

$$\ln EP_{\mathrm{it}}=\rho \sum_{j=1}^{N}W_{ij}\ln EP_{jt}+\rho 0+\rho_{1}DE_{it}+\rho_{2}\sum_{j=1}^{N}W_{ij}DE_{jt}+\rho 3\sum X_{it}^{'}+\rho_{4}\sum_{j=1}^{N}W_{ij}X_{jt}^{'}+\mu_{i}+\gamma_{t}+\varepsilon_{it}$$ (2)

Where, *W*_*ij*_ is an element of the spatial weight matrix, which represents the spatial proximity between cities. *X*’ represents a set of control variables. *μ* is the individual fixed effect. *γ* is the time fixed effect. *ε* is the random error term.

SDM assumes that the variables have a spatial autocorrelation. The observation value of a region is affected by the observation value of the surrounding area. When the variables have a spatial correlation, using this model for empirical analysis not only helps us analyze the spatial spillover effect of variables, but also reduces the deviation of model estimation.

### 4.3 Variable description

This paper selects 267 prefecture-level cities in China from 2012 to 2021 as research samples. The limitation of data set is that the research data is only until 2021, and the current and future relationship between DE and environmental pollution may not be accurately reflected. The explained variable is the environmental pollution index calculated by entropy weight method. The core explanatory variable is the DE index calculated by PCA method [54]. All variables are derived from China City Yearbook, China prefecture-level City Statistical Annual Report and Peking University Digital Finance Center. The environmental pollution, economic development level and population density are logarithmically processed to reduce the impact of outliers. Due to the existence of missing variables and two-way causality, there may be endogenous problems between DE and environmental pollution. In order to solve this problem, we use the interaction term between the number of post offices and fixed telephones owned by cities in 1984 and the number of Internet users nationwide during the sample period as the instrumental variable of DE, and use the instrumental variable method to re-estimate the impact of DE on environmental pollution. See “5.4.6 endogenous problems” for specific practices. Descriptive statistics of all variables are shown in Table 1.

**Table 1:** Descriptive statistical results.

Variable	Definition	N	Mean	SD	Min	Max
**lnEP**	Environmental pollution index	2670	-2.8049	1.6698	-8.6616	0.5960
**DE**	Digital economy index	2670	-1.4690	2.2749	-8.5386	1.2061
**lnpgdp**	Economic development level	2670	10.8277	0.5476	9.2618	12.4108
**lnpop**	Population density	2670	5.8136	0.9158	2.2482	8.8812
**hum**	Human capital level	2670	1.0842	0.6056	0.1888	5.4910
**fdi**	Foreign direct investment	2670	0.0180	0.0256	0.0000	0.7340
**er**	Environmental regulation level	2670	0.0038	0.0019	0.0002	0.0203

#### 4.3.1 Explained variable

Environmental pollution is caused by a variety of pollutants. It is limited to replace environmental pollution with a single pollutant discharge. Thus, the improved entropy weight method is used to evaluate the degree of environmental pollution. Industrial sulfur dioxide emissions, industrial smoke and dust emissions and wastewater emissions are used to construct a comprehensive environmental pollution index. Since the units of each indicator are different, we standardize each indicator to scientifically measure the weight of each indicator [55]. The formula is as follows:

$$x_{ij}^{'}=\frac{x_{ij}-\min(x_{1j},\cdots ,x_{nj})}{max(x_{1j},\cdots ,x_{\mathrm{nj}})-\min(x_{1j},\cdots ,x_{nj})}+1$$ (3)

Where, *x*_*ij*_ represents the value of index *j* of city *i*. *n* is the number of samples. Then, the ratio of index *j* of city *i* to index *j* of all cities is calculated:

$$p_{\mathrm{ij}}=x_{ij}^{'}/\sum_{i=1}^{n}x_{ij}^{'}$$ (4)

According to Formula (4), the entropy of index *j* can be determined as:

$$e_{j}=-k\sum_{i=1}^{n}p_{ij}\ln(p_{ij})$$ (5)

Where, *k* = 1/ln(*n*). According to *e*_*j*_, the weight of the *j* index can be calculated:

$$w_{j}=(1-e_{j})/\sum_{j=1}^{m}\left(1-e_{j}\right)$$ (6)

Where, *m* is the number of environmental pollution sub-indicators. According to Eqs (4) and (6), the environmental pollution index can be expressed as:

$$\mathrm{EP}=\sum_{j}^{m}w_{j}p_{ij}$$ (7)

**Fig 2:**
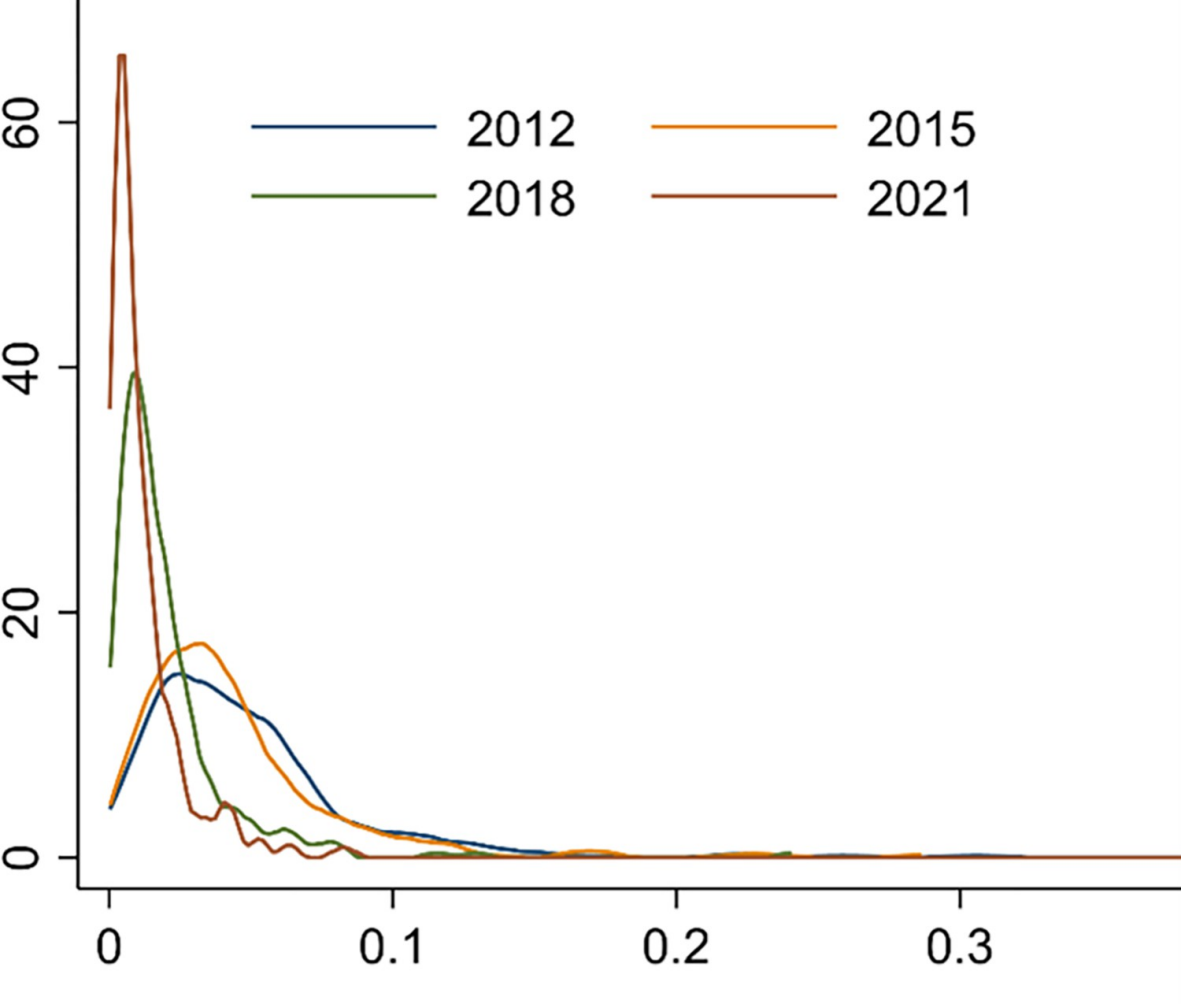
Change trend of environmental pollution degree.

In order to alleviate heteroscedasticity, the environmental pollution index is treated logarithmically. Fig 2 shows the variation trend of environmental pollution levels in China. The level of urban environmental pollution shows a downward trend from 2012 to 2021. The differences of pollution between cities are growing.

### 4.3.2 Explanatory variable

Based on existing studies, this paper constructs a comprehensive index of China’s city-level DE from three aspects: Digital infrastructure, input-output of digital elements and development of digital transactions. Among them, the digital infrastructure is measured by the number of Internet access households per ten thousand people and the number of mobile phones per ten thousand people. Input of digital factors is expressed as the proportion of employees in information transmission, computer services and software industries to total employees, and the proportion of scientific expenditure to GDP. Digital factor output is expressed by per capita output value of telecom industry. The development of digital transactions is measured by the Peking University Digital Financial Inclusion Index. Table 2 shows the evaluation system of DE index. In this study, PCA method is utilized to reduce the dimensions of the above indicators to obtain the DE development index.

**Table 2:** Comprehensive index system of urban DE level.

Primary index	Secondary index	Tertiary index	Unit	Index attribute
**Digital economy index**	Digital infrastructure	Internet access per 10,000 people	Households/10,000 people	+
		Number of mobile phones per 10000 people	Set/10,100 people	+
	Digital factor input and output	The proportion of information transmission, computer service and software industry employees to the employees of urban units	%	+
		The ratio of science expenditure to GDP	%	+
		Per capita telecom industry output	yuan	+
	Digital transaction development	Digital Financial Inclusion Index		+

**Fig 3:**
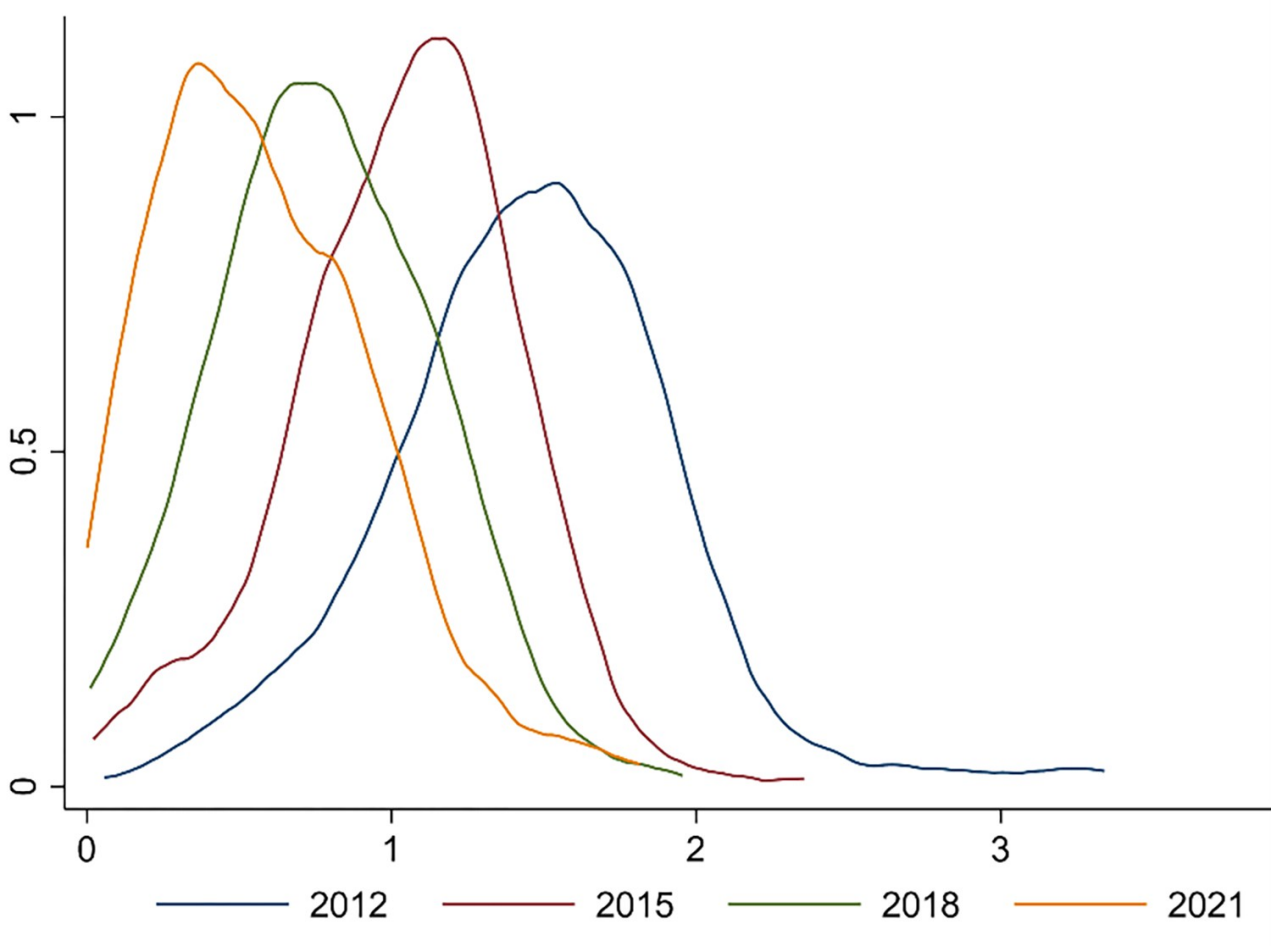
Change trend of DE level.

Fig 3 shows the changing trend of China’s DE index. Chinese cities show an increase in DE levels from 2012 to 2021. Differences in the DE between cities are narrowing.

#### 4.3.3 Control variables

In order to better study the impact of DE on environmental pollution, five control variables are added to the regression model:

1. Economic development level (lnpgdp): China’s traditional development model relies on energy and environmental sacrifice to develop the economy. Therefore, high level of economic development brings serious pollution [56]. However, China’s economy is transitioning to high-quality growth. The economy will develop in a greener direction [57]. This paper chooses per capita GDP to represent the level of economic development.
2. Population density (lnpop): Population density affects environmental pollution mainly through agglomeration effect and scale effect [58]. This study selects the number of population per unit area to represent population density.
3. Human capital (hum): People with higher education levels tend to have stronger environmental awareness and ability, thus reducing pollution emissions [59]. This paper uses the ratio of college students to measure human capital.
4. Foreign direct investment (fdi): Foreign direct investment can bring advanced technologies and produce technology spillovers, thereby reducing pollution [60]. However, some scholars think that foreign direct investment is helpful for the development of pollution-intensive industries in the host country [61]. In this paper, the proportion of foreign direct investment to GDP is selected to evaluate foreign direct investment.
5. Environmental Regulation (er): This paper uses the proportion of government investment in pollution control to GDP to estimate environmental regulation.

#### 4.3.4 Spatial weight matrix

In this paper, geographical distance weight matrix and geographic economy nested matrix are used as spatial weight matrix. The geographical distance weight matrix reflects the law that the correlation between variables decreases with the increase of distance. The geographic economic nested matrix not only considers the geographical distribution, but also considers the degree of economic exchanges between cities.

This paper constructs the most common geographical distance weight matrix. The specific formula is as follows:

$$W{\left(geo\right)}_{ij}=\{\begin{array}{c} 1/d_{ij},i\neq j\\ 0,i=j \end{array}$$ (8)

Where, *d*_*ij*_ indicates the geographical distance between city *i* and city *j*.

Because of the spatial correlation between regions with similar levels of economic development, this paper constructs a nested matrix of geographic economy. The specific formula is as follows:

$$W{\left(geo-\mathrm{eco}\right)}_{ij}=\{\begin{array}{c} (1/d_{ij})*(1/|pgdp_{i}-pgdp_{j}|),i\neq j\\ 0,i=j \end{array}$$ (9)

Where, *d*_*ij*_ has the same meaning as above. $\bar{pgdp_{i}}$ represents the average per capita GDP of city *i* during the sample period. $\bar{pgdp_{j}}$ represents the average GDP per capita of city *j* during the sample period.

## 5 The empirical result analysis

### 5.1 Benchmark regression results

According to Formula (1), the regression without considering spatial effect is carried out to preliminarily explore the relationship between DE and environmental pollution. Table 3 shows the regression results of pooled OLS, time fixed effect model, individual fixed effect model and time and individual double fixed effects model. The coefficients of DE are all significantly negative, which preliminarily determines that environment pollution is negatively impacted by DE. Next, we further explore the impact of DE on environmental pollution considering spatial spillover effect.

**Table 3:** Baseline regression results.

	(1)	(2)	(3)	(4)
**DE**	-0.617***	-0.217***	-0.867***	-0.807***
	(-6.12)	(-9.40)	(-10.92)	(-5.70)
**lnpgdp**	0.173***	0.237***	-1.965***	-0.164**
	(4.35)	(6.81)	(-28.73)	(-1.97)
**lnpop**	-0.0058	0.0056	-1.411***	-1.532***
	(-0.25)	(0.28)	(-5.38)	(-7.19)
**hum**	-0.509***	-0.281***	-0.963***	-0.0781*
	(-14.06)	(-8.87)	(-23.39)	(-1.75)
**fdi**	0.242	0.0084	1.118*	-0.795
	(0.31)	(0.01)	(1.86)	(-1.61)
**er**	10.56	30.76***	22.33***	30.44***
	(0.97)	(3.29)	(2.96)	(4.92)
**cons**	-5.048***	-5.532***	26.34***	7.536***
	(-12.55)	(-15.50)	(16.89)	(5.27)
**City**	YES	YES	YES	YES
**Year**	YES	YES	YES	YES
**r** ^**2**^	0.6322	0.0722	0.5012	0.9286
**N**	2670	2670	2670	2670

Notes: ***, **, and * indicates statistical significance at 1%, 5% and 10%, respectively. The value in parentheses represent t statistics.

### 5.2 Spatial correlation test

Passing spatial correlation test is the premise of using spatial econometric model. In this paper, Moran index is used to test the global and local spatial correlation of environmental pollution. Table 4 shows that the Moran index of environmental pollution from 2012 to 2021 is all significant at the level of 0.01, which means that environmental pollution in a city correlates positively with its surroundings.

**Table 4:** Global Moran’s index of environmental pollution.

	W(geo)			W(geo-eco)		
year	I	Z	P-value	I	Z	P-value
**2012**	0.047	8.293	0.000	0.089	3.040	0.002
**2013**	0.054	9.518	0.000	0.103	3.496	0.001
**2014**	0.057	9.924	0.000	0.116	3.937	0.000
**2015**	0.057	9.923	0.000	0.111	3.768	0.000
**2016**	0.052	9.038	0.000	0.135	4.538	0.000
**2017**	0.054	9.312	0.000	0.153	5.092	0.000
**2018**	0.056	9.706	0.000	0.157	5.216	0.000
**2019**	0.057	9.874	0.000	0.163	5.415	0.000
**2020**	0.058	9.992	0.000	0.166	5.531	0.000
**2021**	0.058	10.068	0.000	0.168	5.582	0.000

**Fig 4:**
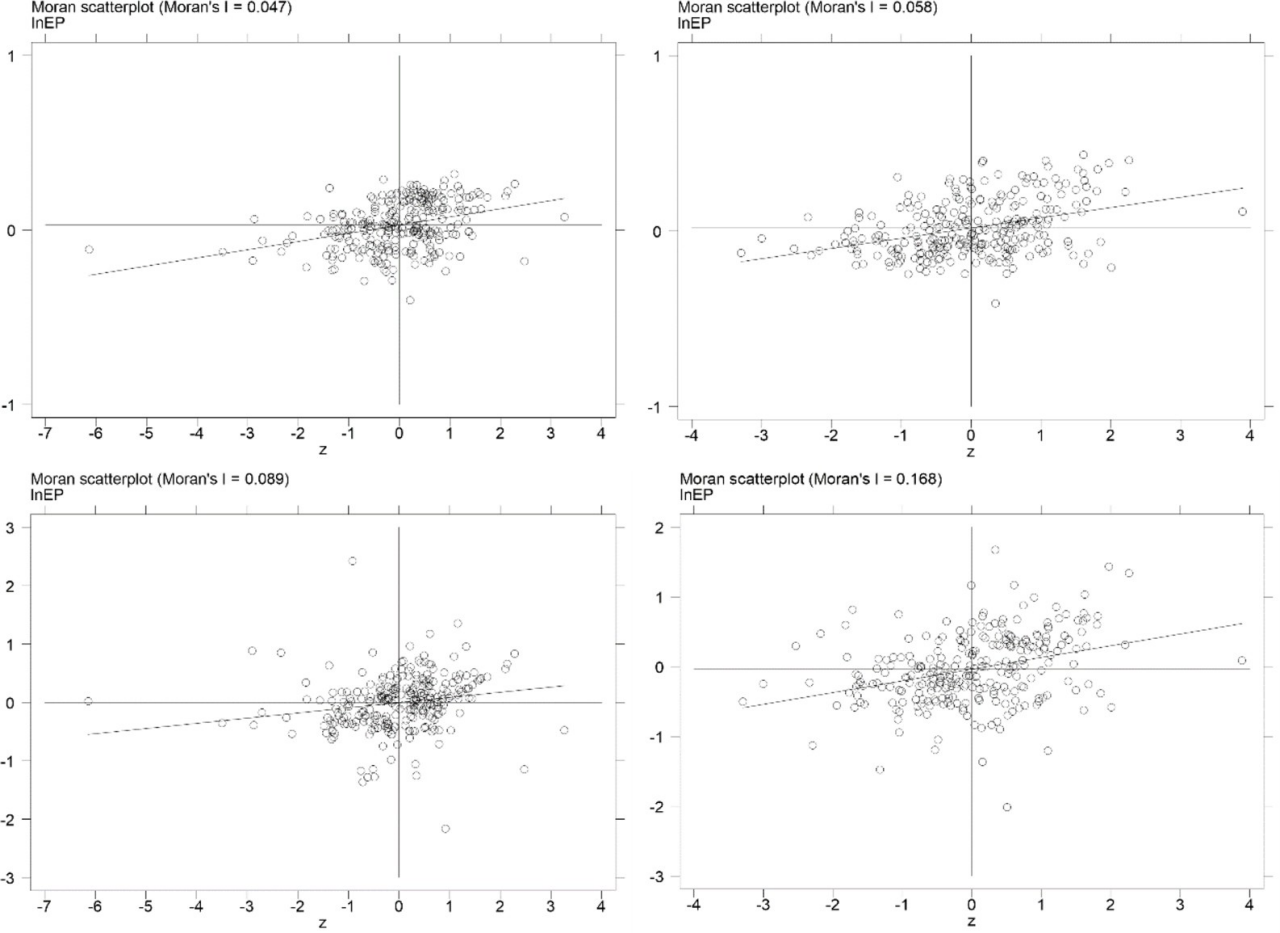
Moran’ s I scatter plots of DE under W(geo) and W(geo-eco). a. 2012_W(geo). b. 2021_W(geo). c. 2012_W(geo-eco). d. 2021_W(geo-eco).

Local Moran scatter plot accurately reflects local spatial correlation. In this paper, the Moran scatter plot of environmental pollution in 267 Chinese cities is plotted. As shown in Fig 4 (Fig 4A–4D), most areas are located in the first and third quadrants under W(geo) and W(geo-eco), which further indicates that there is a positive correlation between environmental pollution.

### 5.3 Spatial econometric model regression results

Before the analysis, it is essential to choose an appropriate econometric model. In this paper, the Lagrange multiplier (LM) is used to determine whether to choose the spatial autoregressive model (SAR), spatial error model (SEM) and SDM. Table 5 shows that LM and robust LM statistics are significant at the 1% level under W(geo) and W(geo-eco). Therefore, SDM cannot be translated into SEM and SAR. This paper chooses SDM, which comprehensively considers spatial panel lag and spatial error lag, to study the impact of DE on environmental pollution.

**Table 5:** Results of the spatial econometric model detection.

	W(geo)		W(geo-eco)	
	Statistic	p-value	Statistic	p-value
**Spatial error:**				
**Moran’s I**	63.277	0.000	19.662	0.000
**Lagrange multiplier**	3453.255	0.000	191.256	0.000
**Robust Lagrange multiplier**	575.334	0.000	30.355	0.000
**Spatial lag:**				
**Lagrange multiplier**	3248.665	0.000	288.364	0.000
**Robust Lagrange multiplier**	277.662	0.000	126.391	0.000

Table 6 reports the results of maximum likelihood estimation based on Formula (2). To make the conclusions more robust, regression results of SAR and SEM models are also reported in this paper. The coefficient of DE is negative at the significance level of 10%, which means that DE can reduce local environmental pollution. The DE, facilitated by the internet and social media, connects a vast number of users, accelerating the dissemination of environmental information. Consumers can directly communicate their demand for eco-friendly products via online channels, driving companies to produce green products. Additionally, DE enables the government to formulate and enforce environmental policies more effectively. Environmental advocates should utilize social media and other digital platforms to spread environmental information and raise public awareness. Companies should produce more eco-friendly and efficient products and services based on consumer feedback, using digital technology to optimize supply chains and reduce emissions during transportation and production. Policy makers should use digital platforms to collect public opinions, allowing citizens to participate in the revision and improvement of environmental policies, thus enhancing their enforcement efficiency.

**Table 6:** Spatial econometric model regression results.

	W(geo)			W(geo-eco)		
	SAR	SEM	SDM	SAR	SEM	SDM
**DE**	-0.0912***	-0.0882**	-0.0704*	-0.0935***	-0.0918***	-0.0665*
	(-2.73)	(-2.51)	(-1.73)	(-2.78)	(-2.65)	(-1.83)
**lnpgdp**	-0.0708	-0.129	-0.345***	-0.0377	-0.0479	-0.113
	(-0.86)	(-1.45)	(-3.48)	(-0.45)	(-0.56)	(-1.26)
**lnpop**	0.0137	0.0063	-0.115	-0.0167	-0.0167	-0.140
	(0.09)	(0.04)	(-0.74)	(-0.11)	(-0.11)	(-0.89)
**hum**	-0.289***	-0.284***	-0.241***	-0.289***	-0.293***	-0.269***
	(-4.10)	(-3.97)	(-3.30)	(-4.05)	(-4.09)	(-3.70)
**fdi**	-1.694**	-1.575*	-0.919	-1.953**	-1.881**	-1.622**
	(-2.05)	(-1.87)	(-1.03)	(-2.38)	(-2.27)	(-1.99)
**er**	-6.519	-5.533	-3.355	-7.828	-7.600	-6.171
	(-1.16)	(-0.98)	(-0.60)	(-1.37)	(-1.33)	(-1.09)
W*DE			-0.396**			-0.178***
			(-2.29)			(-2.96)
W*lnpgdp			3.038***			0.545***
			(4.18)			(2.66)
W*lnpop			-3.244*			0.0781
			(-1.81)			(0.25)
W*hum			0.425			-0.0250
			(0.55)			(-0.14)
W*fdi			-23.08***			-4.811*
			(-3.10)			(-1.93)
W*er			-113.9			-12.10
			(-1.58)			(-0.79)
**Spatial**	0.800***		0.690***	0.156***		0.114**
**rho**	(10.86)		(6.58)	(3.37)		(2.39)
**City**	YES	YES	YES	YES	YES	YES
**Year**	YES	YES	YES	YES	YES	YES
**r** ^**2**^	0.5801	0.3620	0.5331	0.2826	0.2656	0.2820
**N**	2670	2670	2670	2670	2670	2670

Notes: ***, **, and * indicates statistical significance at 1%, 5% and 10%, respectively. The value in parentheses represent t statistics.

The coefficients of W*DE are negative at the 5% and 1% significance levels, respectively, which means that the DE can also reduce environmental pollution in neighboring areas. DE fosters the exchange of knowledge and technology between regions, enhancing the green innovation capabilities of neighboring areas. It also stimulates mutual learning and competition among governments, leading to improved environmental quality in the surrounding regions. Environmental advocates should use digital platforms to promote successful environmental practices from neighboring areas, raising public environmental motivation. Companies should proactively adopt advanced digital technologies and management experiences from adjacent regions to optimize production processes. Additionally, strengthening collaboration with enterprises in neighboring areas to co-develop green products is essential. For policy makers, it’s crucial to learn from the successful cases in neighboring regions where the DE has advanced environmental protection, and to formulate and refine corresponding environmental policies accordingly.

According to Table 7, the direct and indirect effects of DE under W(geo) and W(geo-eco) are significantly negative, which indicates that DE not only reduces the local environmental pollution but also the environmental pollution in the adjacent areas. The result verifies the robustness of regression.

**Table 7:** Direct, indirect and total effects.

	Direct effect		Indirect effect		Total effect	
	W(geo)	W(geo-eco)	W(geo)	W(geo-eco)	W(geo)	W(geo-eco)
**DE**	-0.0761*	-0.0682**	-1.689*	-0.203***	-1.765*	-0.271***
	(-1.69)	(-1.97)	(-1.84)	(-2.98)	(-1.94)	(-3.49)
**lnpgdp**	-0.308***	-0.109	10.58	0.586***	10.28	0.477**
	(-3.00)	(-1.27)	(0.93)	(2.69)	(0.90)	(2.25)
**lnpop**	-0.146	-0.122	-11.95	0.102	-12.09	-0.0204
	(-0.92)	(-0.81)	(-0.88)	(0.30)	(-0.88)	(-0.05)
**hum**	-0.239***	-0.271***	1.046	-0.0524	0.807	-0.293
	(-3.46)	(-3.90)	(0.34)	(-0.26)	(0.27)	(-1.43)
**fdi**	-1.225	-1.685**	-89.40	-5.578**	-90.62	-7.263**
	(-1.44)	(-2.16)	(-1.21)	(-2.06)	(-1.23)	(-2.56)
**er**	-4.565	-5.992	-406.5	-12.72	-411.1	-18.71
	(-0.79)	(-1.06)	(-0.91)	(-0.71)	(-0.91)	(-0.95)

Notes: ***, **, and * indicates statistical significance at 1%, 5% and 10%, respectively. The value in parentheses represent t statistics.

### 5.4 Robustness test

#### 5.4.1 Municipalities are excluded

**Fig 5:**
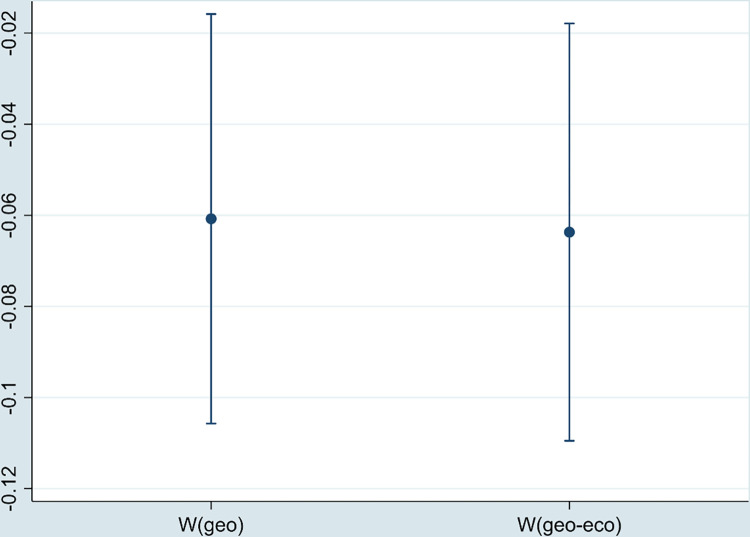
The regression coefficient of DE (excluding municipalities).

As the center of regional economy, municipalities have abundant resources and policy dividends. We eliminate the samples of four municipalities and carry out regression again. As shown in Fig 5, the estimated coefficient of DE is significantly negative, which is consistent with the above results.

#### 5.4.2 Core explanatory variables are replaced

**Fig 6:**
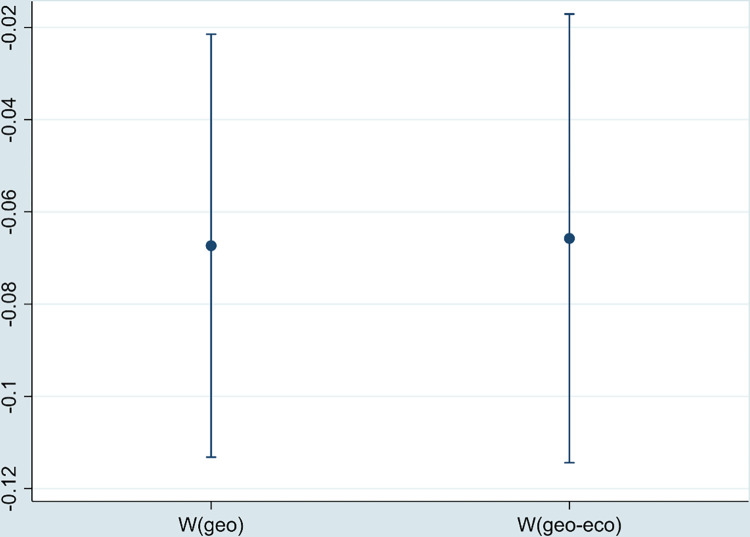
The regression coefficient of DE (replacing the core explanatory variable).

This paper re-calculates DE using the entropy weight method and performs the regression. Fig 6 shows that DE still has a significant negative impact on environmental pollution.

#### 5.4.3 Tail reduction treatment

**Fig 7:**
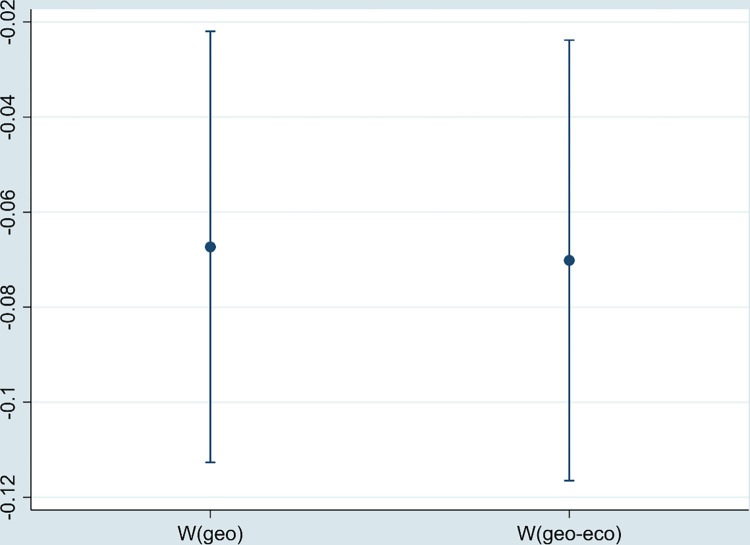
The regression coefficient of DE (tailed processing).

In order to avoid the impact of outliers in the data on the regression results, the sample is treated with 1% bilateral tail reduction. All variables less than the first percentile are replaced with the first percentile. All variables greater than the 99th percentile are replaced with the 99th percentile. As shown in Fig 7, the regression coefficient of DE is still significantly negative.

#### 5.4.4 Sample is replaced

**Fig 8:**
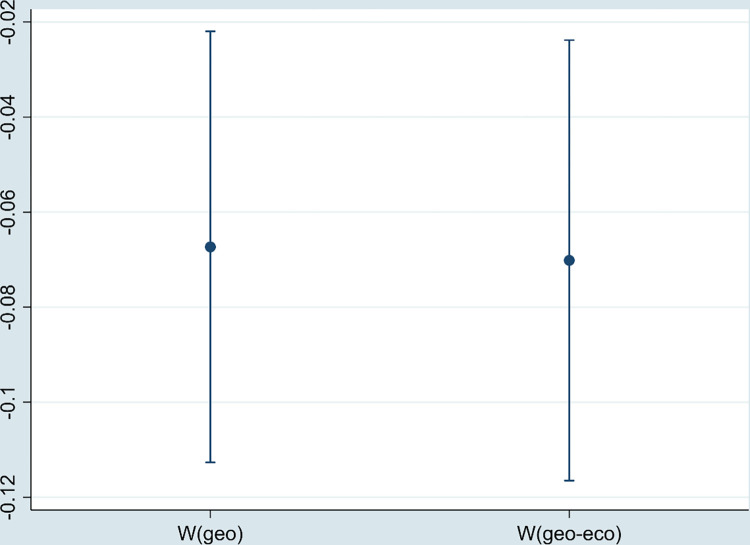
The regression coefficient of DE (replacing sample).

Considering the potential impact of COVID-19 on the estimation results, this paper selects panel data from 267 cities in China from 2011 to 2019 as the research sample to re-estimate the impact of the DE on environmental pollution. As shown in Fig 8, the regression coefficient of DE is still significantly negative.

#### 5.4.5 Exogenous shock test

In this paper, the “Broadband China” pilot policy is used as an exogenous policy shock, and the Difference in Difference (DID) is used as an evaluation method for robustness test. The specific formula is:

$$\begin{array}{l} \ln EP_{\mathrm{it}}=\kappa \sum_{j=1}^{N}W_{ij}\ln EP_{jt}+\kappa_{0}+\kappa_{1}policy_{it}+\kappa_{2}\sum_{j=1}^{N}W_{ij}policy_{jt}\\ \;+\kappa 3\sum X_{it}^{'}+\kappa_{4}\sum_{j=1}^{N}W_{ij}X_{jt}^{'}+\mu_{i}+\gamma_{t}+\varepsilon_{it} \end{array}$$ (10)

Among them, *policy* represents whether it is included in the “Broadband China” pilot list. If it is included, *policy = 1*, otherwise *policy = 0*.

**Fig 9:**
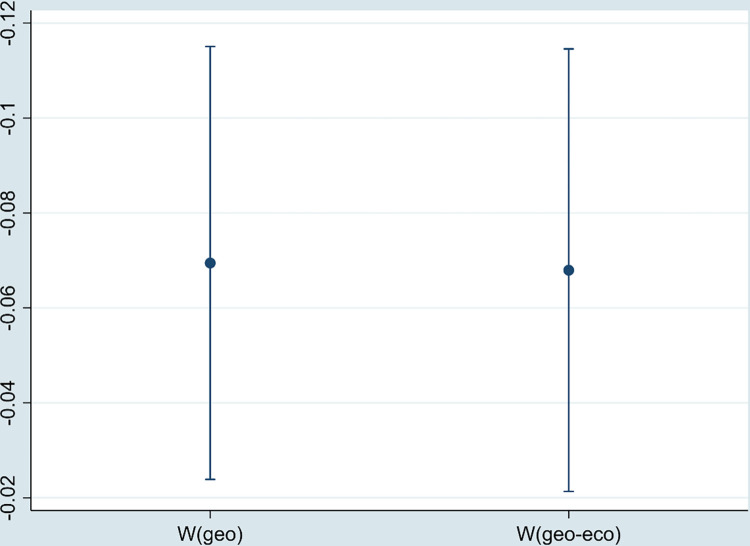
The regression coefficient of “Broadband China”.

Fig 9 shows that the “Broadband China” pilot has a significant negative impact on environmental pollution, which further proves the robustness of empirical results.

#### 5.4.6 Endogeneity problem

Selecting the number of post offices and telephones owned by each city in 1984 as the instrumental variable, this paper uses the instrumental variable method to solve endogenous problems. The use of telecommunications infrastructure affects the application of subsequent Internet technologies in terms of technical level and usage habits, which satisfies the correlation conditions of instrumental variables. Meanwhile, the effect of post office and fixed telephone on environmental pollution decreases with the decrease of the frequency of use until it disappears, which meets the exclusive conditions of instrumental variables. Since the number of post offices and telephones owned by cities in 1984 is cross-sectional data, it cannot be used for panel data analysis. This paper introduces the number of Internet users in the previous year, which changes with time. The interaction term between the number of post offices and telephones owned by cities in 1984 and the number of Internet users during the sample period is used as an instrumental variable for DE.

Table 8 shows that after considering endogenous problems, the impact of DE on environmental pollution is still significantly negative. In addition, the test results prove the validity and rationality of instrumental variables.

**Table 8:** Estimation results of instrumental variable method.

Instrumental variables	POS		PHO	
	(1)	(2)	(3)	(4)
DE		-0.292**		-0.758**
		(-2.42)		(-2.38)
lnpgdp	-0.0684	-0.0725	-0.2161**	-0.111
	(-0.77)	(-0.61)	(-2.56)	(-0.78)
lnpop	-0.5100**	-0.0775	-0.8017***	-0.307
	(-2.15)	(-0.41)	(-3.67)	(-0.97)
hum	0.0593	-0.299**	-0.0305	-0.274***
	(0.75)	(-2.55)	(0.41)	(-2.76)
fdi	0.5214	-2.303*	0.4591	-1.860**
	(0.50)	(-1.90)	(-0.53)	(-2.10)
er	4.3808	-7.584	1.2185*	-3.703
	(0.65)	(-1.07)	(1.79)	(-0.50)
iv	0.2319***		0.7669***	
	(4.38)		(2.99)	
City	Yes	Yes	Yes	Yes
Year	Yes	Yes	Yes	Yes
r^2^	0.4180	0.6928	0.4291	0.6336
N	2670	2670	2670	2670
F test	19.17***		8.97***	
Kleibergen-Paap rk LM statistic		19.302***		8.721***
Cragg-Donald Wald F statistic		19.167		8.969
Stock-Yogo bias critical value		16.38(10%)		8.96(15%)

Notes: ***, **, and * indicates statistical significance at 1%, 5% and 10%, respectively. The value in parentheses represent t statistics.

## 6 Further analysis

### 6.1 Transmission mechanism

#### 6.1.1 Mediation effect model

The main assumption of mediation effect model is that there is a clear causal relationship. The explanatory variable affects mediating variable, and the mediating variable further affects explained variable. It is mainly used to understand the logical relationship between explanatory variable and explained variable more deeply. According to the analysis, DE may affect environmental pollution through industrial structure upgrading and green technology innovation. The mediation effect model established in this paper is as follows:

$$\ln EP_{\mathrm{it}}=\eta_{0}+\eta_{1}DE_{it}+\eta_{2}\sum X_{it}^{'}+\mu_{i}+\gamma_{t}+\varepsilon_{it}$$ (11)

$$M_{\mathrm{it}}=\theta_{0}+\theta_{1}DE_{it}+\theta_{2}\sum X_{it}^{'}+\mu_{i}+\gamma_{t}+\varepsilon_{it}$$ (12)

$$\ln EP_{\mathrm{it}}=\lambda_{0}+\lambda_{1}DE_{it}+\lambda_{2}M_{\mathrm{it}}+\lambda_{3}\sum X_{it}^{'}+\mu_{i}+\gamma_{t}+\varepsilon_{it}$$ (13)

Where, *M* is the intermediary variable, represented by industrial structure upgrading and green technology innovation. The industrial structure upgrading is measured by the ratio of the added value of the tertiary industry to that of the secondary industry. Green technology innovation is measured as the logarithm of the number of green patents produced plus one. If the coefficient *η*_1_, *θ*_1_ and *λ*_2_ are significant, and the coefficient *λ*_1_ is less than *η*_1_, it means that the mediating variable has a mediating effect to associate DE and environmental pollution.

#### 6.1.2 Industrial structure upgrading

Column (1) of Table 9 shows that the coefficient of DE is negative at the significance level of 1%, which means that DE has a negative influence on environmental pollution. As shown in column (2) of Table 9, the coefficient of DE is positive at the significance level of 5%, indicating that DE also promotes the upgrading of industrial structure. In column (3) of Table 9, both DE and industrial structure upgrading are included in the regression model. The coefficients of DE and industrial structure upgrading are significantly negative. The DE has a negative effect on environmental pollution by guiding the upgrading of industrial structure, which verifies the first half of hypothesis 2. DE promotes the development of capital and knowledge-intensive industries, leading to an upgrade in the industrial structure. In this advanced industrial structure, high-tech industries like information and biotechnology become dominant, characterized by low pollution emissions. Enterprises should proactively invest in R&D of digital technologies and transform their industries into more sustainable production models. For policy makers, it’s vital to provide financial support and tax incentives to enterprises to encourage the development of digital technologies and industrial transformation, aiming to reduce pollution emissions.

**Table 9:** Results of mechanism verification.

	(1)	(2)	(3)	(4)	(5)
	lnEP	isu	lnEP	lngi	lnEP
**DE**	-0.807***	0.3245**	-0.7056***	0.1641**	-0.7707***
	(-5.70)	(2.28)	(-4.08)	(2.33)	(-4.85)
**isu**			-0.3124***		
			(-2.96)		
**lngi**					-0.221**
					(-2.25)
**cons**	7.536***	5.2675***	6.7844***	-2.4067	6.8709***
	(5.27)	(3.73)	(3.92)	(-1.40)	(4.00)
**Control**	YES	YES	YES	YES	YES
**City**	YES	YES	YES	YES	YES
**Year**	YES	YES	YES	YES	YES
**r** ^**2**^	0.9286	0.7221	0.8625	0.7414	0.8894
**N**	2670	2670	2670	2670	2670

Notes: ***, **, and * indicates statistical significance at 1%, 5% and 10%, respectively. The value in parentheses represent t statistics.

#### 6.1.3 Green technology innovation

Based on theoretical analysis, this paper examines the mediating effect of green technology innovation in the relationship between DE and environmental pollution. Column (4) in Table 9 shows that the coefficient of DE is positive at the significance level of 5%, indicating that DE has a significant positive impact on the degree of green technology innovation. According to column (5) of Table 9, the coefficients of both DE and green technology innovation are significantly negative. To sum up, DE can reduce environmental pollution through green technology innovation, which verifies the second half of hypothesis 2. As a significant application of DE, digital finance lowers the threshold for businesses to access funding, providing more resources for green technology R&D. Additionally, DE encourages the exchange and collaboration in green technology innovation among enterprises, enhancing green technological advancements.

For enterprises, it’s essential to actively utilize digital platforms to bolster communication and collaboration with other businesses in green technology. This collaboration propels green technology innovation and reduces pollution emissions during production. Policy makers should establish public digital platforms to support the innovation and information sharing of green technologies, promoting their development and application.

### 6.2 Threshold effect test

#### 6.2.1 Panel threshold model

Panel threshold model is based on the assumption of nonlinear relationship. It is assumed that the relationship between the explained variable and explanatory variable changes with the change of threshold variable. The model is mainly used to test the nonlinear relationship between explanatory variables and explained variables. In order to further analyze the effect of DE on environmental pollution under different marketization degrees, this paper constructs the following panel threshold model:

$$\begin{array}{l} \ln EP_{\mathrm{it}}=\chi_{0}+\chi_{1}DE_{it}I(MARKit<\delta 1)+\chi 2DE_{it}I(\delta 1\leq MARKit<\delta 2)\\ \;+\chi 3DE_{it}I(MARKit\geq \delta 2)+\chi 4\sum X_{it}^{'}+\mu_{i}+\gamma_{t}+\varepsilon_{it} \end{array}$$ (14)

Among them, *MARK* is the threshold variable marketization degree. *δ* is the threshold value, and *δ*_1_<*δ*_2_. *I*(⋅) is an exponential function, when the condition is met, *I*(⋅) = 1. Otherwise, *I*(⋅) = 1.

#### 6.2.2 Threshold effect test

According to theoretical analysis, the emission reduction effect of DE is affected by the degree of marketization. This paper uses Bootstrap repeated sampling method to estimate the threshold value of marketization degree. Table 10 shows that there is a single threshold effect in the degree of marketization. The threshold value is 11.6611.

**Table 10:** Threshold effect test results.

Number of thresholds	Threshold values	Fstat	Prob	10%	5%	1%
**Single threshold**	11.6611	18.11	0.0500	13.6497	17.3640	26.6192
**Double threshold**		4.19	0.7433	13.7534	15.9574	21.1622

**Fig 10:**
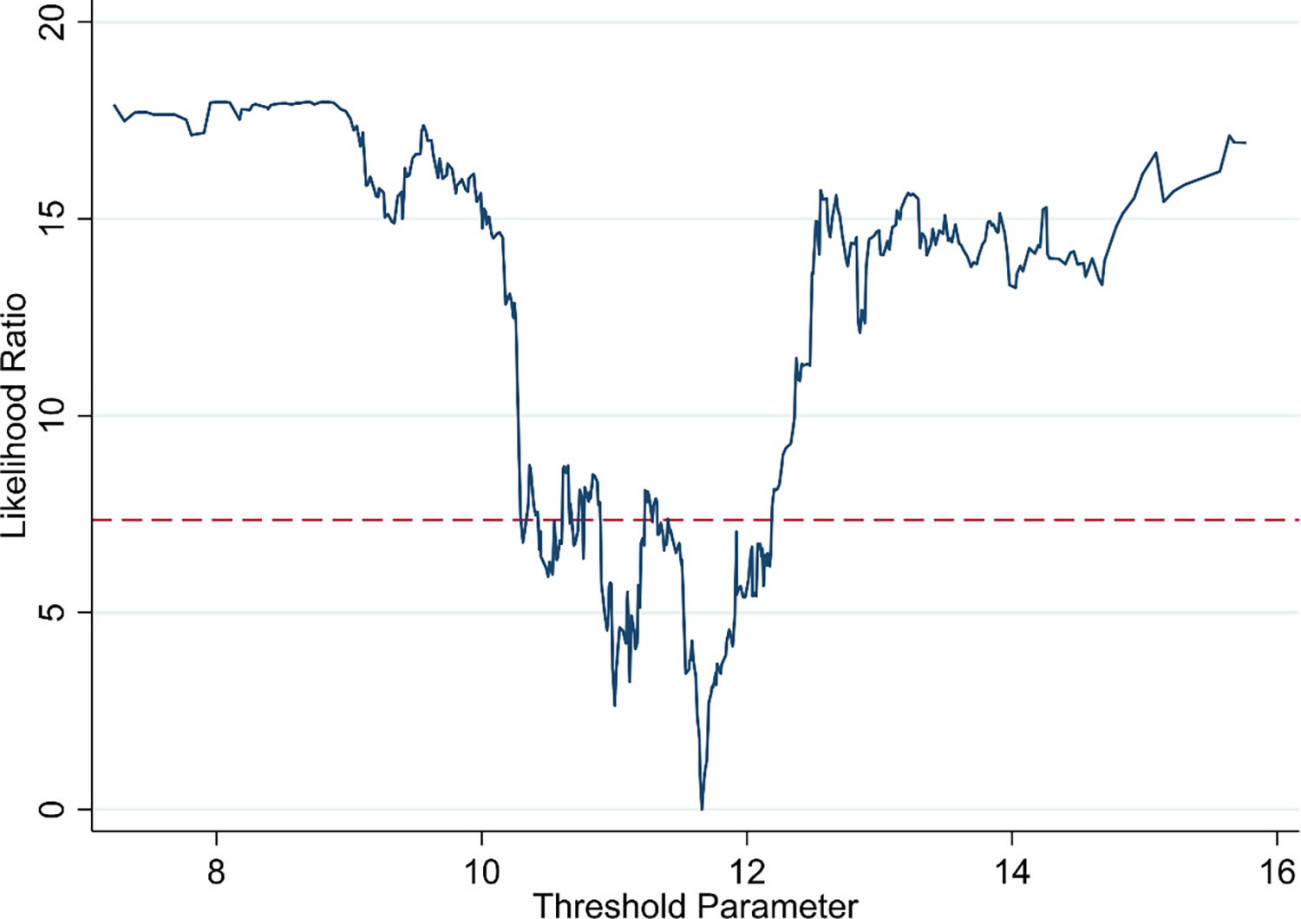
Threshold value and confidence interval.

This paper draws Fig 10 to intuitively reflect the threshold value and confidence interval of marketization degree. The dotted line represents the critical value of the likelihood ratio statistic. The interval of all likelihood ratio values less than the critical value at the 5% significance level is the confidence interval.

#### 6.2.3 Panel threshold model regression results

Table 11 shows that when the degree of marketization is below the threshold, the estimated coefficient of DE is -0.0042, but not significant. The possible reason are as follows: When the degree of marketization is not high and government intervention is dominant, the role of market mechanism in optimizing resource allocation efficiency is difficult to play. Production factors related to DE cannot flow to related industries according to market orientation. Therefore, the DE is difficult to play a role in emission reduction. When the degree of marketization crosses the threshold, the estimated coefficient of DE is -0.111 and passes the 1% significance level. With the continuous improvement of marketization, the allocation efficiency of factors has been improved, thus promoting the emission reduction effect of DE. As the degree of marketization increases, the emission reduction effects of DE are enhanced. The advancement of marketization reduces entry barriers to DE, increases the number of low-pollution enterprises. Intense market competition drives companies to develop clean production technologies, thereby reducing pollutant emissions. Simultaneously, increased marketization enhances multi-directional information flow and the demonstration effect of pollution control, improving regional environmental quality.

**Table 11:** Panel threshold model regression results.

	Coefficient	T value
**DE I(MARK≤11.6611)**	-0.0042	-0.14
**DE I(MARK>11.6611)**	-0.111***	-4.11
**lnpgdp**	-0.0852	-0.92
**lnpop**	-0.0250	-0.15
**hum**	-0.305***	-4.29
**fdi**	-1.883**	-2.12
**er**	-8.137	-1.30
**cons**	-1.973	-1.36
**r** ^**2**^	0.6927	
**N**	2670	

Notes: ***, **, and * indicates statistical significance at 1%, 5% and 10%, respectively. The value in parentheses represent t statistics.

Enterprises should swiftly adapt to market changes, utilize digital technologies to optimize production processes, and enhance resource-use efficiency. Policy makers should further promote market openness, lower the entry barriers to DE, and allow a flourish of more green production technologies.

### 6.3 Heterogeneity analysis

#### 6.3.1 Geographical location heterogeneity

Considering the differences in institutional environment and geographical conditions in different regions, the DE has a regional differential influence on environmental pollution. This paper divides the samples into eastern cities, central cities and western cities according to geographical location and carries out regression again.

Table 12 shows that the DE only has a negative impact on environmental pollution in eastern and central cities. The DE can better leverage the advantages of resource integration and reduce pollutant emissions in the east and center of China. The western region, however, is in the early stages of digital infrastructure construction. The digital industry causes about as much environmental pollution as it reduces emissions.

**Table 12:** Regression results of heterogeneity.

	Eastern cities	Central cities	Western cities	Resource- based city	Non resource- based city	Central city	Non-central city
**DE**	-0.1325***	-0.1377**	-0.0624	-0.1761**	-0.0715**	-0.1659***	-0.0720**
	(-3.06)	(-2.02)	(-0.82)	(-2.30)	(-2.42)	(-3.59)	(-2.33)
**cons**	-11.0376***	0.3625	7.3885	-2.7222	-1.6512	-9.25**	-2.3556
	(-3.199)	(0.65)	(1.21)	(-1.54)	(-0.76)	(-1.98)	(-1.34)
**Control**	YES	YES	YES	YES	YES	YES	YES
**City**	YES	YES	YES	YES	YES	YES	YES
**Year**	YES	YES	YES	YES	YES	YES	YES
**r** ^**2**^	0.6882	0.7048	0.6881	0.6326	0.6533	0.8105	0.6233
**N**	1130	1080	460	1070	1120	330	2340

Notes: ***, **, and * indicates statistical significance at 1%, 5% and 10%, respectively. The value in parentheses represent t statistics.

#### 6.3.2 Resources endowment heterogeneity

According to the “*Notice of The State Council on Printing and Distributing the National Sustainable Development Plan for Resource-Based Cities (2013–2020)*”, the samples are grouped into resource-based cities and non-resource-based cities for re-regression.

Table 12 shows that whether in resource-based cities or non-resource-based cities, DE has a negative effect on environmental pollution. The negative impact is even greater in resource-based cities. Resource-based cities are rich in natural resources. Compared with non-resource-based cities, the development and processing activities of resource-based cities have a worse impact on the environment. DE in resource-based city has a greater marginal effect on energy conservation and emissions reduction. As a city rich in coal, limestone and other resources, the coal production of Tongchuan in Shaanxi Province accounts for 70% of Shaanxi Province. In recent years, Tongchuan City insists on the development of DE as a leading project. It has built the province’s first “5G+ optical network” double-gigabit commercial city. Jingdong, iFlytek and more than 20 other leading enterprises settles in Tongchuan. The number of DE market entities has reached more than 600. E-commerce transactions exceeds 20 billion yuan. With the change of industrial mode, the improvement rate of PM2.5 in Tongchuan City is 16.3% in 2021, which is the first in China.

#### 6.3.3 Administrative level heterogeneity

According to China’s administrative system, 267 cities are divided into 33 central cities (including municipalities, sub-provincial cities and provincial capitals) and 234 non-central cities. The heterogeneous impact of DE on environmental pollution at different administrative levels is further analyzed in this paper.

Table 12 shows that the negative impact of DE on environmental pollution in central cities is greater than that in non-central cities. The possible reasons mainly include the following three aspects: Firstly, compared with non-central cities, central cities can better integrate the DE with real economy, thereby expanding the impact of the DE on environmental pollution. Secondly, the implementation process of administrative orders in central cities is convenient, which promotes the optimal allocation of resources. The emerging industry of DE can grow better, thus expanding the impact of DE on environmental pollution. Thirdly, central cities attach great importance to DE and pollution control policies, which also expands the impact of DE on environmental pollution.

## 7 Discussion

Environmental pollution levels can be reduced through DE. Firstly, in the age of DE, by spreading information and knowledge rapidly people become more aware of the environment. Secondly, DE can prevent unnecessarily wasted resources due to a mismatch between supply and demand. Thirdly, the DE can reduce unnecessary activities, thus reducing environmental pollution. Meanwhile, DE can reduce environmental pollution through industrial structure upgrading and green technology innovation. As for the upgrading of industrial structure, resource allocation can be made more efficient with DE, which leads to an upgrade of industrial structure. The upgrading of industrial structure can reduce energy consumption and pollutant emission. As for green technology innovation, DE can improve the efficiency of green technology resource integration and environmental monitoring capacity. By using green technology, production efficiency can be improved and pollution levels can be reduced. In recent years, Anyang City of Henan Province has vigorously promoted the development of DE and accelerated the construction of digital infrastructure, which has provided strong support for the development of DE. In 2021, the improvement rate of Anyang’s comprehensive environmental index ranks first in Henan Province and 9th among 168 cities in China.

DE has a significant negative spatial spillover effect on environmental pollution. Firstly, DE can promote inter-regional exchange and interaction, and generate knowledge and technology spillover effect. Secondly, the DE can provide capital, labor and other means of production to neighboring regions. Thirdly, the interaction and competition between governments also reduce the level of environmental pollution. The Yangtze River Delta is the most developed city cluster of DE in China. According to the *Yangtze River Delta Digital Economy Development Report (2021)*, the scale of DE in the Yangtze River Delta accounts for about 44% of the regional GDP in 2020, and about 28% of the total scale of the national DE. In 2020, the number of days with good air quality in 41 cities in the Yangtze River Delta is 85.2 percent, up 8.7 percentage points, which is higher than the national average improvement rate.

Thirdly, the improvement of marketization can enhance the emission reduction effect of DE. The possible reasons are as follows: Firstly, the improvement of marketization can accelerate the free flow of production factors. More capital flows to environmentally friendly and efficient industries, thereby reducing pollutant emissions. Secondly, the improvement of marketization can also intensify market competition and force enterprises to carry out clean technology innovation. Thirdly, the improvement of marketization enables information between enterprises to flow to multiple fields. The demonstration effect of successful pollution control improves the environmental quality across the entire region. In May 2014, taking the decisive role of market in the allocation of resources as a starting point, Fujian introduced the emission trading system. The introduction of this system has prompted new projects to adopt more advanced technologies to reduce pollutant emissions, which achieves source emission reduction. Meanwhile, it also has prompted enterprises to take more stringent pollution control measures, which achieves end treatment.

## 8 Conclusions and policy recommendations

Based on previous studies, this paper theoretically examines the effect mechanism of DE on environmental pollution, and puts forward four research hypotheses. The empirical results of this study prove the validity of research hypothesis. Specifically, based on panel data from 267 Chinese cities from 2012 to 2021, this paper empirically examines DE’s influence on environmental pollution and its mechanism by using SDM, intermediary effect model and panel threshold model. Considering the differences in geographical location, resource endowment and administrative level, we divide the entire sample into eastern, central, and western regions, resource-based and non-resource-based cities, central cities and non-central cities. This paper analyzes the impact of DE on the heterogeneity of environmental pollution in cities with different geographic locations, resource endowments and administrative level. The conclusions are as follows: (1) DE can effectively decrease the degree of environmental pollution. (2) DE reduces environmental pollution mainly through industrial structure upgrading and green technology innovation. (3) DE has negative spatial spillover effect on environmental pollution. DE can not only decrease the level of local environmental pollution, but also decrease the level of environmental pollutants in neighboring areas. (4) DE has obvious market-oriented single threshold effect on environmental pollution. When the marketization level is lower than 11.6611, the impact of DE on environmental pollution is not significant. When the marketization level crosses 11.6611, the emission reduction effect of DE gradually appears. (5) DE has heterogeneous impacts on environmental pollution in cities with different geographical locations, resource endowments and administrative level. In terms of geographical heterogeneity, environmental pollution is significantly reduced in east and middle of China by DE, but not in western China. In terms of resource endowments, compared with non-resource-based cities, the DE in resource-based cities has more emission reduction effect due to their heterogeneous resource endowments. In terms of administrative level, the emission reduction effect of DE in central cities is greater than that in non-central cities. The following policy recommendations are made in light of the above conclusions:

1. Strengthening the application of DE in emission reduction. The government should actively use data analysis technology to analyze pollution data, thereby formulating targeted environmental protection policies. Enterprises should use DE to optimize the production process, improve production efficiency, and reduce environmental pollution. Additionally, enterprises should use digital platforms to strengthen cooperation with other companies, facilitating the sharing of resources like green technology and knowledge.
2. Regarding the upgrading of industrial structure, the government should provide financial support and tax incentives to encourage more companies to transition to digital and low-pollution production modes. A flexible policy environment should be constructed to reduce the cost for enterprises to use digital technology for industrial transformation. Companies should utilize digital technology to obtain market information and promptly adjust their industrial development models to be more environmentally friendly. Regarding green technology innovation, the government should actively organize digital skill training activities, making more innovators use digital technology to promote green technology innovation. Enterprises should use digital platforms to strengthen connections with other green technology developers, enhancing their own green technology innovation capabilities.
3. Strengthen cooperation and exchanges between regions. DE has a negative spatial spillover effect on environmental pollution. Therefore, efficient and convenient digital platforms should be used among cities to strengthen technological cooperation and exchange, so as to achieve coordinated emission reduction among cities. Simultaneously, a number of other measures should be taken simultaneously by government, including improving the DE governance system and strengthening the protection of intellectual property to prevent knowledge and technology spillover from happening to the country. In addition, the government should learn from nearby areas’ successful use of DE in reducing emissions and accordingly develop and enhance its environmental protection policies.
4. Enhancing the degree of marketization. As the level of marketization increases, the emission reduction effect of DE gradually strengthens. The government should reduce excessive regulation to provide enterprises with more free space for development. Enterprises should construct flexible and adaptive business strategies to quickly adapt to market changes. Utilizing digital technology to optimize production processes, thereby increasing production efficiency and reducing environmental pollution.
5. Formulate relevant DE policies according to specific conditions. Western regions are not significantly affected by DE emission reduction, unlike eastern and central areas. Because the infrastructure of DE in the western region is not perfect enough, forming a digital gap with developed regions, which limits the rapid development of DE in the western areas. Therefore, the government should give some policy support to the western areas. Investment in DE infrastructure is increased in the western region. The digital divide is narrowed. The dividends of the development of the DE are fully released.

## Supporting information

S1 File (XLSX)

S2 File Graphical summary. (DOCX)
