# Supplementary figures and images for: The impact of digital economy on environmental pollution: Evidence from 267 cities in China
Source: PLoS One. 2024 Jan 26;19(1):e0297009. doi: 10.1371/journal.pone.0297009 (PMC10817194; doi:10.1371/journal.pone.0297009)

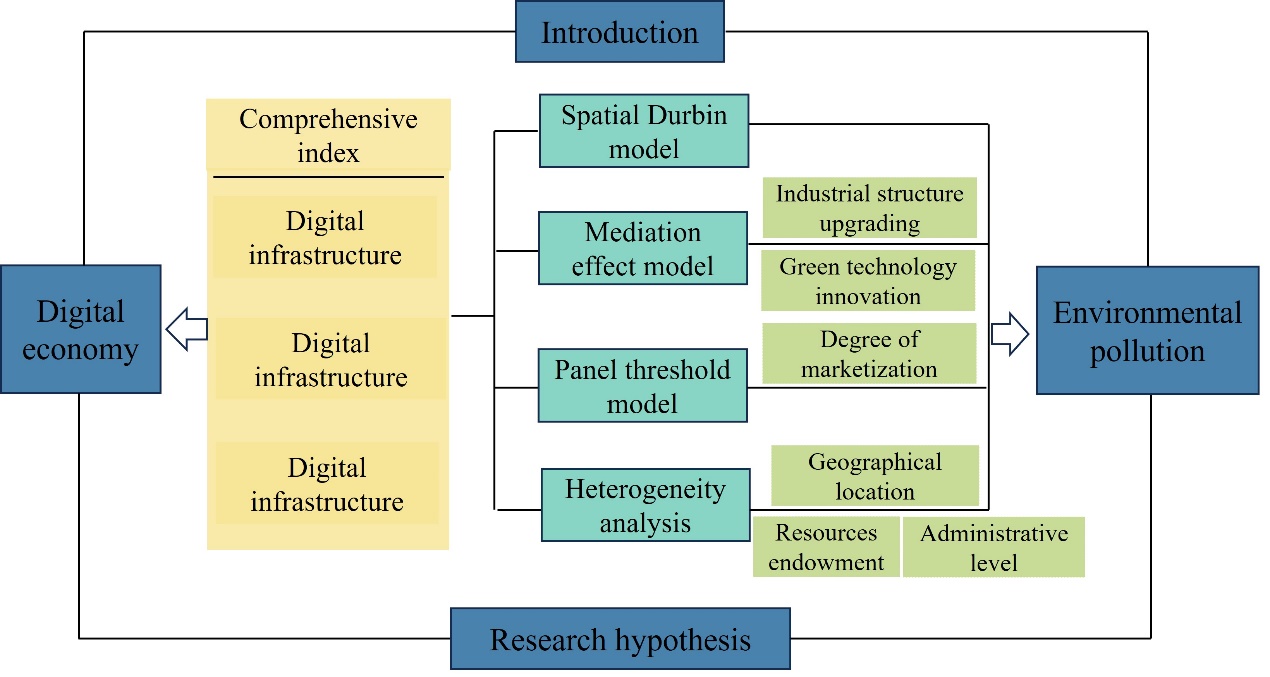


**S1 Fig. Graphical summary**

Supplement: S2 File — (DOCX) [file pone.0297009.s002.docx]
